# Salivary Microbial Dysbiosis Is Associated With Peri-Implantitis: A Case-Control Study in a Brazilian Population

**DOI:** 10.3389/fcimb.2021.696432

**Published:** 2022-01-05

**Authors:** Debora Pallos, Vanessa Sousa, Magda Feres, Belen Retamal-Valdes, Tsute Chen, Mike Curtis, Richardson Mondego Boaventura, Marcia Hiromi Tanaka, Gustavo Vargas da Silva Salomão, Louise Zanella, Tania Regina Tozetto-Mendoza, Gabriela Schwab, Lucas Augusto Moyses Franco, Ester Cerdeira Sabino, Paulo Henrique Braz-Silva, Jamil Awad Shibli

**Affiliations:** ^1^Department of Dentistry, University of Santo Amaro, São Paulo, Brazil; ^2^Centre for Oral Clinical Research, Centre for Oral Immunobiology & Regenerative Medicine, Institute of Dentistry, Barts and The London School of Medicine and Dentistry, Queen Mary University London, London, United Kingdom; ^3^Department of Periodontology and Oral Implantology, Dental Research Division, Guarulhos University, Guarulhos, Brazil; ^4^Department of Oral Medicine, Infection & Immunity, Harvard School of Dental Medicine, Boston, MA, United States; ^5^Dental Institute, King’s College London, Guy’s Hospital Tower Wing, London, United Kingdom; ^6^Department of Stomatology, School of Dentistry, University of São Paulo, São Paulo, Brazil; ^7^Laboratory of Integrative Biology (LIBi), Scientific and Technological Bioresource Nucleus—Center for Excellence in Translational Medicine (BIOREN–CEMT), Universidad de La Frontera, Temuco, Chile; ^8^Institute of Tropical Medicine of São Paulo, School of Medicine, University of São Paulo, São Paulo, Brazil

**Keywords:** peri-implantitis, saliva, microbiota (16S), host–bacteria interaction, dysbioses

## Abstract

**Background and Objectives:**

The aim of this study was to examine the salivary microbiome in healthy peri-implant sites and those with peri-implantitis.

**Methods:**

Saliva samples were collected from 21 participants with healthy peri-implant sites and 21 participants with peri-implantitis. The V4 hypervariable region of the 16S rRNA gene was sequenced using the Ion Torrent PGM System (Ion 318™ Chip v2 400). The NGS analysis and composition of the salivary microbiome were determined by taxonomy assignment. Downstream bioinformatic analyses were performed in QIIME (v 1.9.1).

**Results:**

Clinical differences according to peri-implant condition status were found. Alpha diversity metrics revealed that the bacterial communities of participants with healthy peri-implant sites tended to have a richer microbial composition than individuals with peri-implantitis. In terms of beta diversity, bleeding on probing (BoP) may influence the microbial diversity. However, no clear partitioning was noted between the salivary microbiome of volunteers with healthy peri-implant sites or volunteers with peri-implantitis. The highest relative abundance of *Stenotrophomonas*, *Enterococcus* and *Leuconostoc* genus, and *Faecalibacterium prausnitzii*, *Haemophilus parainfluenzae*, *Prevotella copri*, *Bacteroides vulgatus*, and *Bacteroides stercoris bacterial species* was found in participants with peri-implantitis when compared with those with healthy peri-implant sites.

**Conclusion:**

Differences in salivary microbiome composition were observed between patients with healthy peri-implant sites and those with peri-implantitis. BoP could affect the diversity (beta diversity) of the salivary microbiome.

## Introduction

The use of dental implants to restore or replace lost tooth structure in partially and completely edentulous subjects is a successful treatment in Dentistry. The market share of implants was valued at US$ 3 billion in 2016 with over 9 million implants placed in Europe, USA, and Brazil (MARCANTONIO JUNIOR et al., 2019). However, the prevalence of peri-implant diseases is growing in the same proportion and ranged between 1% and 47% (Lee et al., 2017; Salvi et al., 2017). Peri-implantitis is a plaque-associated pathological condition, characterized by inflammation of the peri-implant tissues associated with bone loss, and if not treated, could lead to loss of the implant (Berglundh et al., 2018; Schwarz et al., 2018). Peri-implant diseases are highly prevalent in subjects with diabetes, smoking cigarettes, and history or presence of periodontitis. The clinical signs of peri-implants are bleeding on probing and/or suppuration, presence of probing pocket depth >5 mm, and radiographic bone loss. The etiology of peri-implantitis has been the subject of some debate; however, since then it has been established that this infectious disease is associated with a complex bacterial biofilm (Shibli et al., 2008; Pérez-Chaparro et al., 2016; Lafaurie et al., 2017; Teles, 2017; Retamal-Valdes et al., 2019; Yeh et al., 2019). As there is continually growing prevalence of this condition, it is of the utmost importance to characterize the specificity of the dental biofilm related to peri-implantitis.

The microbial diversity of the submucosal and salivary microbiome associated with peri-implantitis has not been fully studied (Belibasakis and Manoil, 2021). Peri-implantitis depicted changes in the submucosal microbiome associated with an increased level of dysbiosis in deeper pockets (Kröger et al., 2018). Some studies have also shown that peri-implant diseases presented higher diversity than clinically healthy peri-implant sites, suggesting that specific species in this pathogenesis could impact in an increasing in the Shannon index (Komatsu et al., 2020; Nie et al., 2020). Complementary in this sense, a recent consensus report considered that the microbial picture associated with peri‐implantitis should be regarded as incomplete (Schwarz et al., 2018). The systematic and detailed evaluation of the microbiota of peri-implantitis, using sequencing techniques, could contribute to the advancement of knowledge in this area and improve the effectiveness of peri-implantitis treatments.

In research, salivary biomarkers have been extensively used as diagnostic tools for the early detection of several oral and systemic diseases (Giannobile et al., 2009; Zhang et al., 2016). The collection of whole saliva is an easy, low-stress, inexpensive, and non-invasive procedure, particularly for chair-side tests, as recently used for SARS-CoV-2 detection (Yokota et al., 2021). Earlier studies have demonstrated that clinical periodontal and peri-implant status data were correlated with salivary biomarkers (Gursoy and Könönen, 2016; Kuboniwa et al., 2016; Marques Filho et al., 2018). In addition, implant-supported restorations with peri-implantitis showed an increased level of salivary periodontal pathogens (Ito et al., 2014), suggesting that salivary biomarkers could be used for monitoring peri-implant diseases in an easy way.

Therefore, the aim of this case–control study was to compare the composition and diversity of the salivary microbiome in samples taken from subjects with healthy peri-implant sites or with peri-implantitis.

## Materials and Methods

### Study Design and Setting

This was a case–control study, conducted at Guarulhos University (Guarulhos, SP, Brazil), in compliance with the principles outlined in the Declaration of Helsinki. The study protocol was previously approved by the Guarulhos University Clinical Research Ethics Committee, Guarulhos, SP, Brazil (protocol number #205/03). A total of 42 subjects (N = 21 subjects with healthy dental implants and n = 21 subjects with diseased implants) were enrolled. The sample size population was based on a previously study that compared supra- and submucosal microbial composition between healthy and diseased implants, by using target molecular methods. Besides the absence of previous studies evaluating the salivary microbiome of peri-implantitis and the sample sizes described in recent studies (Apatzidou et al., 2017; Belkacemi et al., 2018; Kröger et al., 2018; Pimentel et al., 2018) that evaluated the microbiome of samples of dental implants from crevicular fluid and inner part, the present sample population was consistent for this case–control study.

### Participants

Systemically healthy volunteers with dental implant-supported restoration in function for at least 2 years and diagnosed with healthy peri-implant sites or peri-implantitis were selected from the population that sought dental treatment at Guarulhos University (Guarulhos, SP, Brazil). Subjects who fulfilled the inclusion criteria were invited to participate in the study. All eligible subjects were thoroughly informed of the nature, potential risks, and benefits of their participation in the study and then signed an Informed Consent Term. Detailed medical and dental histories were obtained, and clinical examinations were performed.

### Inclusion and Exclusion Criteria

The inclusion criteria for the study groups were as follows: (i) *group with healthy peri-implant sites*: subjects with at least one healthy dental implant (probing depth [PD] < 4 mm, without marginal bleeding or bleeding on probing [BoP]) and (ii) *group with peri-implantitis*: presence of BOP and/or suppuration, PD ≥ 5 mm, and bone loss ≥3 mm apical of the most coronal portion of the intraosseous part of the implant (Shibli et al., 2008).

The exclusion criteria were as follows: mucositis (implants with PD ≤ 4 mm, supramucosal bleeding, without bone loss); periodontitis grade 3 (i.e., PD ≥ 5 mm, BOP in over 30% of sites and/or suppuration); those who had taken antibiotics or anti-inflammatory drugs within 6 months prior to the clinical examination; those who had received periodontal or peri-implant therapy within 6 months; those who had a chronic medical disease or condition (i.e., diabetes, osteoporosis); those who presented implant-supported restoration with mobile abutments and/or screws, and fractured prosthetic crowns made of ceramic or resin (to avoid occlusal interference); those who had clinically detectable mobility of the implant (lack of osseointegration); and smokers and former smokers.

### Experimental Design

#### Clinical Monitoring and Dental Implant Selection

Dichotomous plaque score (PS) (Guerrero et al., 2005), bleeding score (BS), BoP, suppuration, PD (in mm), and clinical attachment level (CAL, in mm) were assessed at six sites per implant by two calibrated examiners (MF; JAS). The PD and CAL measurements were recorded to the nearest mm using a North Carolina periodontal probe (PCPNU-15, Hu-Friedy, Chicago, IL, USA). One dental implant was evaluated (one per subject). If the subject presented more than one healthy dental implant, the most anterior dental implant was evaluated. All subjects from the diseased group presented only one dental implant with peri-implantitis. If the subject presented healthy and diseased implants, he was included in the peri-implantitis group, and the diseased implant was evaluated.

##### Calibration Exercise

The calibration exercise was conducted before the study began, according to the methodology proposed by Araujo et al. (2003). The interexaminer variability was 0.3 mm for PD and 0.3 mm for CAL. For the first examiner, the intraexaminer mean SE variability was 0.1 mm for PD and 0.1 mm for CAL. The second examiner had a mean SE variability of 0.20 mm and 0.22 mm for PD and CAL, respectively. The periodontal parameters were registered dichotomously; that is, plaque accumulation, gingival bleeding, BoP, and suppuration were calculated in the same way, with two different evaluations by the k-light test (p < 0.05), which considers the contribution of agreement by chance. The interobserver agreement ranged between 0.85 and 0.95, while the intraobserver agreement was between 0.80 and 0.96 for the first examiner and 0.80 and 0.87 for the second examiner.

#### Microbiological Monitoring

##### Sample Collection

Saliva samples were collected on a different day from that of the clinical examination. Study participants were asked to refrain from eating, drinking, or performing oral hygiene for 12 h prior to biological sampling. Unstimulated whole saliva that had accumulated for 5 min was collected in sterile plastic tubes labeled with codes to ensure concealment of the patients’ identity (Navazesh and Christensen, 1982). Sample tubes were subsequently stored immediately at -20°C for further 16S rRNA gene sequencing analysis at the Institute of Tropical Medicine of São Paulo (University of São Paulo, São Paulo, Brazil).

##### DNA Isolation

Total genomic DNA was extracted and purified (NucliSENS easyMAG system, and Beckman Coulter™ Agencourt AMPure XP), and quality and quantity of DNA were assessed.

##### 16S rRNA Sequencing

The V4 hypervariable region of bacterial and archaeal species of the 16S rRNA gene was amplified using a specific primer with the barcode F515 (5′-CACGGTCGKCGGCGCCATT-3) and R806 (5′-GGACTACHVGGGTWTCTAAT-3′) (Caporaso et al., 2012). Library preparation was performed in accordance with the manufacturer’s instructions (*Ion PGM™ Hi-Q™*). The 16S rRNA gene fragments were loaded onto an Ion Torrent PGM System chip (Life Technologies, USA) and sequenced using Ion 318™ Chip kit v2 400-base chemistry.

### Bioinformatic and Statistical Methods

The 16S rRNA raw sequencing readouts were analyzed using the BLAST-Based Open-Reference 16S rRNA NGS Species-Level Read Assignment pipeline (Acharya et al., 2019). Briefly, 16S rRNA read numbers were BLASTN-searched against a combined set of 16S rRNA reference sequences that consist of the HOMD (version 14.51), HOMD 16S rRNA RefSeq Extended Version 1.1 (EXT), GreenGene Gold (GG), and the NCBI 16S rRNA reference sequence set. Readouts with ≥98% sequence identity to the matched reference and ≥98% alignment length were classified based on the taxonomy of the reference sequence with the highest sequence identity. If a read matched the reference sequences representing multiple species equally (i.e., equal percent identity and alignment length), it was submitted to chimera checking. Non-chimeric reads with multispecies best hits were considered valid and were assigned as a different species with multiple species names. Unassigned reads (i.e., ≤98% identity or ≤98% alignment length) were pooled together and subjected to the *de novo* chimera checking and sequence quality screening using the USEARCH program version v8.1.1861. The *de novo* chimera checking was done using 98% as the sequence identity cutoff. Non-chimeric unassigned reads that were ≥200 bases were then subjected to species-level *de novo* operational taxonomy units, so-called amplicon sequence variants (ASV) with 98% as the sequence identity cutoff using USEARCH. Representative reads of each of the ASV/species were BLASTN-searched against the same reference sequence set again, to determine the closest species for these potential novel species.

Samples with <500 read counts were excluded from the QIIME analysis. Therefore, two samples (b1Peri17 and b6Peri14) were removed from the QIIME analysis due to having low numbers of sequences (<500 sequences). Raw sequencing readouts belonging to 40 samples were analyzed using the BLAST-Based Open-Reference 16S rRNA NGS Species-Level Read Assignment pipeline (Acharya et al., 2019). The phylogenetic tree required for constructing the UniFrac-based matrices used in some of the beta diversity analyses was built dynamically from reference sequences with matched reads. The reference sequences were aligned with the software MAFFT version 7.149b prior to tree construction using the QIIME treeing script. Downstream analyses were made for a range of minimal read counts (MC) per ASV/species (MC = 100). All assigned reads were subject to several downstream bioinformatics analyses, including alpha and beta diversity assessments, provided in the QIIME software package (version 1.9.1). Alpha diversity analyses included the Shannon diversity index that measures both richness and evenness (increases as there are more evenly distributed taxa) and the Simpson diversity index (1-D) that is higher when communities are more diverse. Beta diversity analyses were performed by means of unweighted UniFrac (Lozupone and Knight, 2005). In addition, in order to visualize the similarity between samples, principal coordinate analysis (PCoA) and fit function *pcoa* were implemented. Group significance (Kruskal–Wallis, p values) and *post-hoc* analyses (FDR and Bonferroni correction) were examined to identify ASVs that differed in abundance between healthy peri-implant sites and those with peri-implantitis. A differential taxonomic representation of the salivary microbiome in the clinical diagnosis of peri-implantitis and healthy peri-implant sites was performed (Metacoder R, 2017) (Foster et al., 2017). Clinical parameters were assessed by Mann–Whitney *U*. The level of significance was set at 5%.

## Results

### Clinical Characteristics

Forty-two volunteers were included in this study, 21 patients with healthy peri-implant sites and 21 patients with peri-implantitis. In the group with healthy peri-implant sites, there were 8 males and 13 females, and the mean age was 49.0 ± 12.2 years [range 27–71 years]. In the groups with peri-implantitis, there were 3 males and 18 females with mean age 52.6 ± 12.1 years [range 28–77 years]. **Table 1** shows that the main clinical parameters of implants included in the study were higher for the peri-implantitis group (p < 0.05; **Table 1**).

**Table 1:** Mean (± SD) clinical parameters for monitoring peri-implant conditions, stratified per group.

Clinical variables	Healthy peri-implant sites (n = 21)	Peri-implantitis (n = 21)	p-value^#^
PD (mm)	3.27 ± 0.99	5.06 ± 2.28	0.0050*
CAL (mm)	0.15 ± 0.64	5.08 ± 2.03	<0.0001*
PS (%)	43.1 ± 43.7	31.7 ± 40.1	0.5058
BS (%)	26.5 ± 35.3	53.9 ± 36.1	0.0227*
BoP (%)	50.0 ± 36.4	80.8 ± 30.8	0.0089*
Suppuration (%)	0 ± 0	10.7 ± 0.1	<0.01*
Bone loss (mm)	1.74 ± 2.90	4.81 ± 2.56	<0.0001*
**Characteristics of the dental implants**			
*Number of* Anterior: posterior	9: 12	8:13	
Screw retained: cement	10: 11	11: 10	
Single units	5	5	
FDP (2 or more units)	12	11	
Fully edentulous (n)	4	6	

PD, pocket depth; CAL, clinical attachment level; PS, plaque score; BS, bleeding score; BoP, bleeding on probing; FDP, fixed dental prosthesis.

^#^Mann–Whitney U (Bold: *p < 0.05).

### Structure and Diversity of the Communities

**Figure 1:**
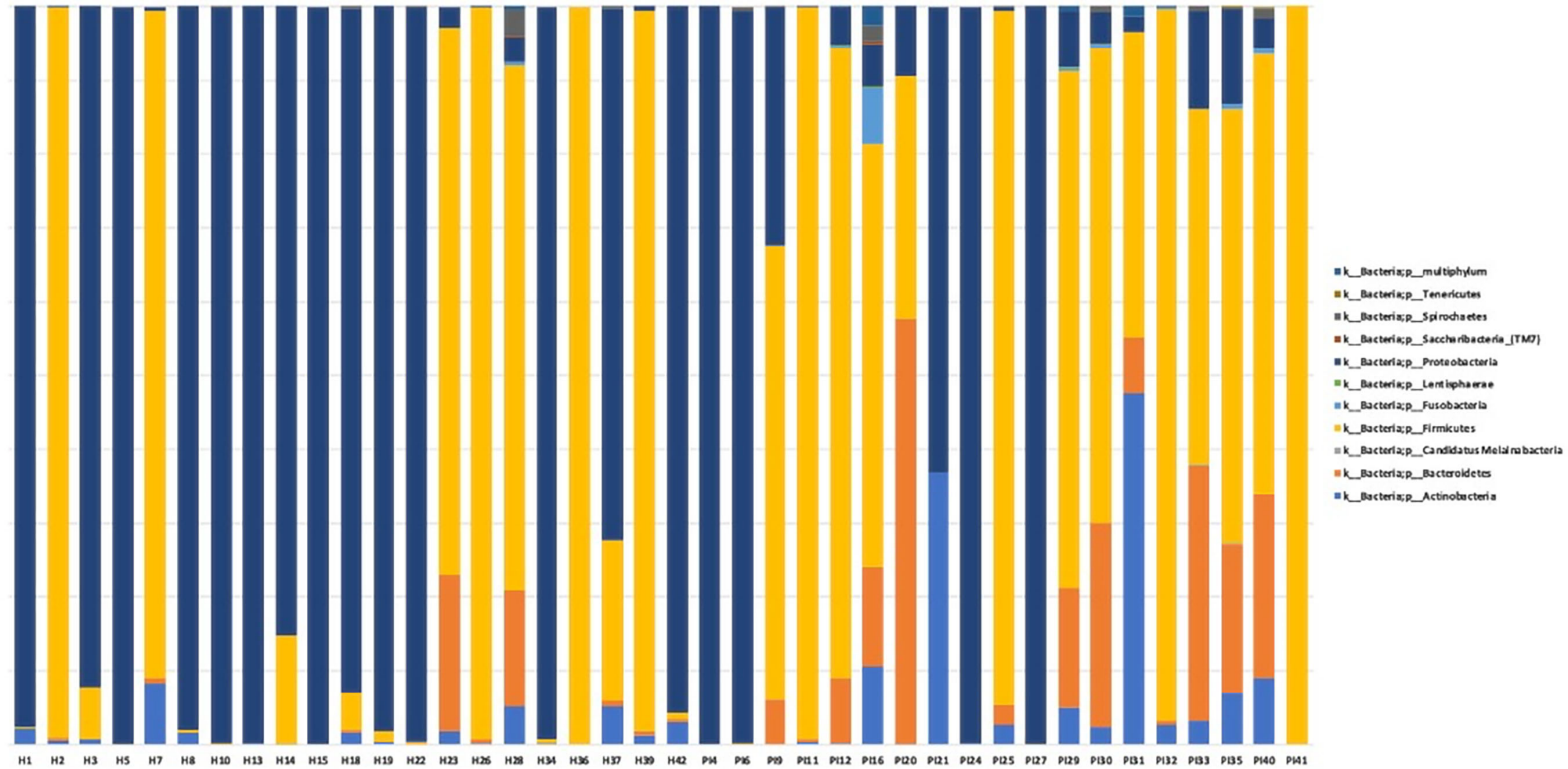
Taxonomic profiles of all relative abundances at a phylum level. Data were normalized and displayed by sample. H, healthy peri-implant site samples; P, peri-implantitis samples.

The total number of assigned reads was 4,993,541 in 40 samples. The total number of ASVs was classified in 11 phyla, 185 genera, and 591 species (MC = 100) (**Figure 1**).

**Figure 2:**
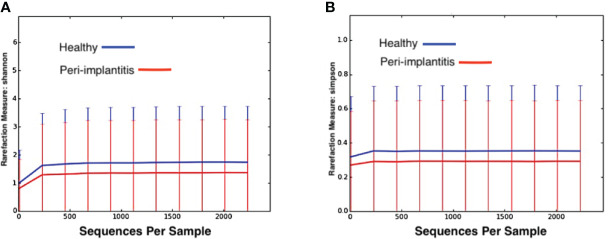
Two measures of alpha diversity in whole saliva samples from groups with healthy peri-implant site (blue) and groups with peri-implantitis (red). **(A)** Shannon index, **(B)** Simpson Diversity Index.

When alpha diversity metrics was applied using Shannon and Simpson indexes (**Figure 2**), it was observed that the bacterial communities of participants with healthy peri-implant sites tended to have richer microbial compositions than those in individuals with peri-implantitis (p > 0.05).

**Figure 3:**
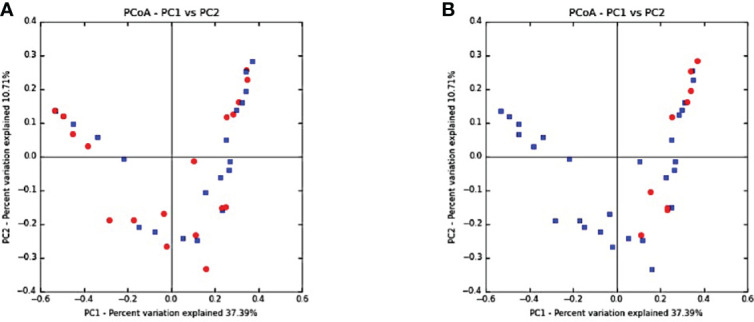
Beta diversity: principal coordinate analysis (PCoA) with Unifrac distance matrices. **(A)** Healthy peri-implant sites (blue) vs. peri-implantitis (red). **(B)** Bleeding on probing (BoP) <10% showing clustering toward the upper and lower quadrants (BoP <10% in red and BoP >10% in blue) in groups with peri-implantitis and those with healthy peri-implant sites.

Beta diversity was analyzed using principal coordinate analysis (PCoA) with Unifrac distance matrices; no clear partitioning was noted between salivary microbiomes of volunteers with healthy peri-implant sites or those with peri-implantitis (**Figure 3A**). However, a similar clustering pattern was observed between samples derived from participants who showed BoP < 10% and BoP > 10% (**Figure 3B**).

**Figure 4:**
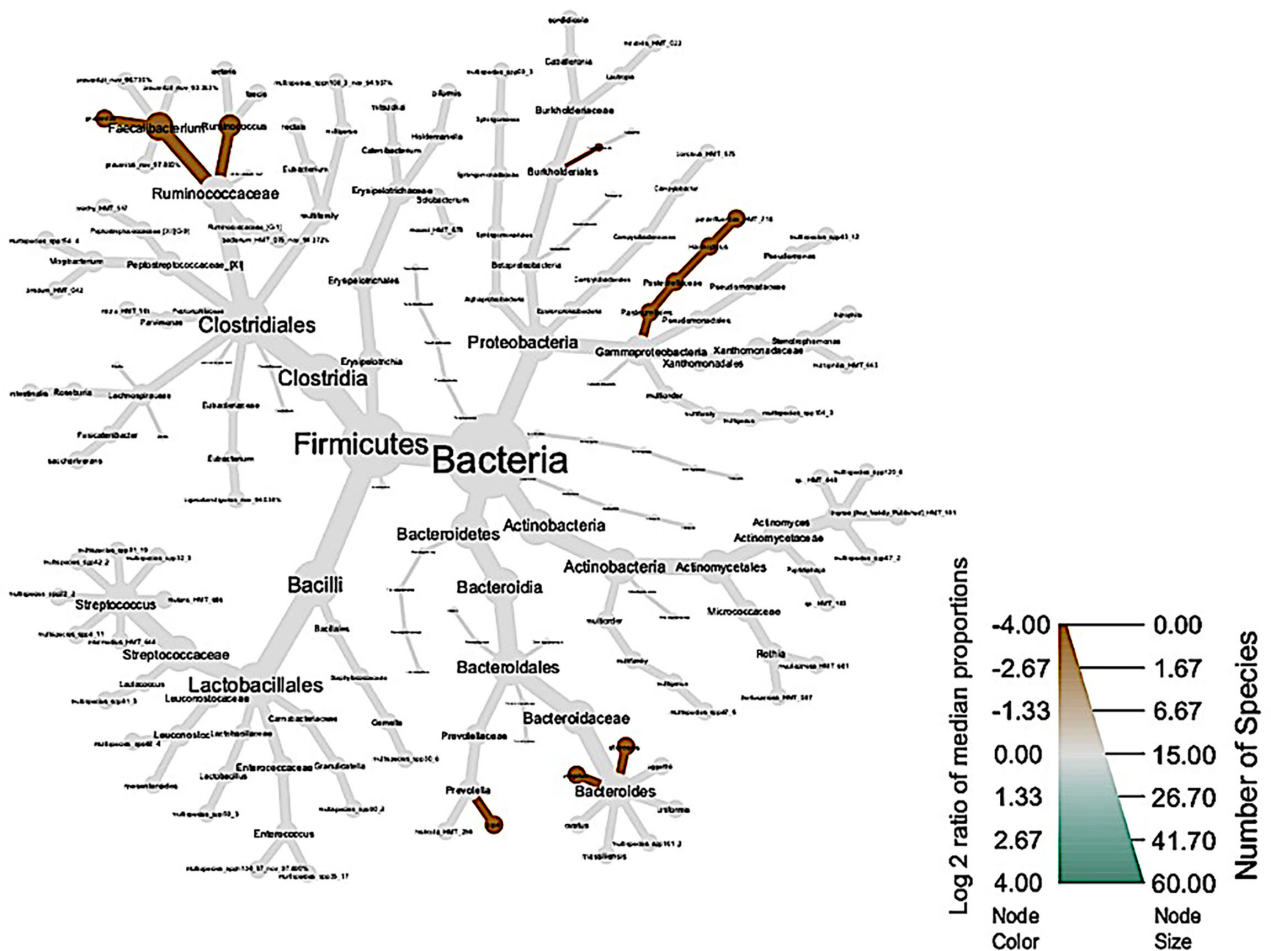
Phylogenetic diversity of salivary microbiome in clinical diagnosis of groups with peri-implantitis (brown) and those with healthy peri-implant sites (green).

The global taxonomic composition of bacterial communities based on the diagnosis of healthy peri-implant sites and those diagnosed with peri-implantitis (**Figure 4**). Healthy subjects did not present phylogenetic diversity, represented by the absence of green color.

### Genus Level

The highest relative abundance in the salivary microbiome of participants with peri-implantitis was represented by the *Stenotrophomonas* (46.2%), *Enterococcus* (11.46%), and *Leuconostoc* (10.17%) genera (SP1). In contrast, the salivary microbiome of volunteers with healthy peri-implant sites exhibited higher relative abundances of *Streptococcus* (6.81%), *Gammaproteobacteria multigenus* (5.25%), and *Actinomyces* (1.56%) genus (SP1).

### Species Level

The following groups of bacteria were detected to be significantly more abundant in conditions of peri-implantitis: *Faecalibacterium prausnitzii*, *Haemophilus parainfluenzae, Prevotella copri*, *Bacteroides vulgatus*, and *Bacteroides stercoris*.

In contrast to the group with peri-implantitis, the salivary microbiome in the group with healthy peri-implant sites exhibited higher levels of abundance of *Rothia mucilaginosa (HMT_681)*, *Haemophilus parainfluenzae (HMT_718)*, and *Actinomyces multispecies (spp120_6)* (SP2).

## Discussion

In this study, a cross-sectional evaluation of the saliva microbiome of subjects with and without peri-implantitis was described for the first time. Overall, the results suggested that there was a difference in the salivary composition of bacterial communities between participants with clinical diagnosis of peri-implantitis and those with healthy peri-implant sites. BoP also showed an effect on diversity (beta diversity) of the salivary microbiome. However, no significant differences were observed in the salivary microbiome richness (alpha diversity) and diversity (beta diversity) between participants with peri-implantitis and those with healthy peri-implant sites.

Saliva has been suggested to be a mirror of the body and could be used to monitor the general health and the onset of specific diseases, including SARS-CoV-2 detection (Giannobile et al., 2009; Yokota et al., 2021). Unfortunately, it has not yet been used in association with the different peri-implant conditions. In fact, the study of the microbiome associated with peri-implantitis when compared with healthy peri-implant sites has focused on characterizing the submucosal biofilm of the participants affected by this disease (Kumar et al., 2012; da Silva et al., 2014; Apatzidou et al., 2017; Sanz-Martin et al., 2017; Al-Ahmad et al., 2018). One study collected gingival crevicular fluid (Gao et al., 2018). The results of these studies have shown that the composition of the peri-implant microbiome differed significantly from that of healthy peri-implant sites. In addition, the biofilm associated with peri-implantitis harbored more pathogenic bacterial species. However, in the study of periodontal diseases, it has been demonstrated that pathogens were located in the entire mouth, not only in deep periodontal pockets (Ximénez-Fyvie et al., 2000; Mager et al., 2003; Haffajee et al., 2008). In this context, saliva could play an important role in the identification of these pathogens.

Our findings, using 16S rRNA-gene sequencing of the salivary microbiome, described the highest relative abundance of the *Stenotrophomonas*, *Enterococcus*, and *Leuconostoc* genera and the *Faecalibacterium prausnitzii*, *Haemophilus parainfluenzae, Prevotella copri*, *Bacteroides vulgatus*, and *Bacteroides stercoris* bacterial species in participants with peri-implantitis when compared with those with healthy peri-implant sites. No recognized pathogens were identified as being relatively abundant in the salivary microbiome of this study group. In fact, based on the authors’ knowledge, these species had not been previously identified in oral samples in the study of the peri-implant microbiota. These findings highlight two important points. First is the importance of including several databases as a comparison parameter in the study of the oral microbiota. If some species had not been found in a microenvironment, it cannot mean that they were not there, but that they had not been the object of study or identification. Secondly, different sample protocols employed in this study, even using saliva, could influence the abundance and quality of the microbiome. In addition, we could speculate that some characteristics of the dental implants, including implant surface topographies, free energy, and material composition, could influence the retention of a mature dental biofilm, mainly during the dysbiosis, and avoid its dispersion in the oral environment through saliva. The microbes may be “protected” in the microgaps, pits, and grooves of the rough surfaces of some implant surface topographies, as titanium plasma-sprayed (TPS) and hydroxylapatite-coated (HA) and anodized surfaces. Therefore, saliva could contain bacterial species, but in less proportion and diversity than in the subgingival microenvironment directly associated with the surface of the dental implant and also of the implant-supported resotration, as was verified at the periodontal level by Mager et al. (2003). They examined the proportions of 40 bacterial species in samples collected from eight oral soft tissue surfaces, saliva, and supra- and subgingival biofilm in patients with periodontitis and those who were periodontally healthy. The authors concluded that the proportions of bacterial species differed markedly on different intraoral surfaces, and the microbiota of saliva was most similar to that of the dorsal and lateral surfaces of the tongue. These findings highlight the importance of the effect of nature of the surface to be colonized on subsequent biofilm composition. This difference in the microbiological profile of saliva samples could also justify the absence of significant differences in the analyzes of alpha and beta diversity when comparing the salivary microbiome in patients with peri-implantitis and in those with healthy peri-implant sites, observed in the present study.

DNA sequencing technologies in dental and periodontal research have revealed the wide diversity of the oral microbiome (Paster et al., 2001). This prompted a new set of studies in oral ecology and the identification of new potential pathogens. Therefore, the identification of this specific group of species and genera associated with healthy peri-implant sites and disease is the starting point for future research in the area.

Although this case–control study has designed to compare the salivary microbiome between healthy and diseased peri-implant sites, a clustering of sites with BoP was associated with the bacterial dysbiosis. The percentage of sites with bleeding on probing influenced the beta-diversity of the salivary microbiome, as presented in **Figure 3**. The members of the red and orange complexes have previously been identified and found to be significantly elevated at sites that exhibited bleeding on probing, which was used as a clinical indicator of periodontal inflammation (Socransky and Haffajee, 2005; Shibli et al., 2008; Kröger et al., 2018). In peri-implant tissues, bone levels ≥3 mm apical of the most coronal portion of the intra‐osseous part of the implant together with BoP were consistent with the diagnosis of peri‐implantitis (Berglundh et al., 2018). Therefore, the clinical characteristics of inflammation at the peri-implant site level may influence the diversity of the salivary microbiome, in agreement with a previous study (Kröger et al., 2018).

This study was the first one to use 16S rRNA gene sequencing to compare the salivary microbiome in volunteers diagnosed with peri-implantitis and those with healthy peri-implant sites. Collecting saliva as a study sample involves relatively simple procedures, and the use of saliva-based oral fluid diagnostics seems promising as a diagnostic criterion for oral diseases. In addition, the development of the chair-side test might facilitate the earlier diagnostic of the microbial shift of implant-supported restorations and therefore improve the treatment plan of the periodontist.

The present study has some strengths as rigorous inclusion criteria (patients without clinical periodontal diseases), sample size, and correlations with clinical parameters. The inclusion and exclusion criteria performed in this study influenced the absence of phylogenic diversity in the salivary microbiome among healthy subjects The main limitation of our study was the lack of microbiological data from subgingival peri-implant sites. The direct comparison between submucosal and salivary samples may lead to criticism of these results as a validated method. However, a better characterization of the peri-implant microbiome can improve the understanding of the etiology of peri-implant diseases and, consequently, improve the treatment of peri-implantitis. Finally, the outcomes presented in this investigation were obtained from a specific population, and therefore, factors such as smoking cigarettes, diabetes, history of periodontitis, different characteristics of the dental implants, and type of implant-supported restoration could modify the results.

In conclusion, the results of this cross-sectional study showed that there was a difference in the salivary composition of bacterial communities between participants with clinical diagnosis of peri-implantitis and those diagnosed with healthy peri-implant sites. BoP could affect the diversity (beta diversity) of the salivary microbiome.

## Data Availability Statement

The datasets presented in this study can be found in online repositories. The names of the repository/repositories and accession number(s) can be found below: NCBI SRA BioProject, accession no: PRJNA747611.

## Ethics Statement

The studies involving human participants were reviewed and approved by the Guarulhos University Clinical Research Ethics Committee. The patients/participants provided their written informed consent to participate in this study.

## Author Contributions

DP, MF, ES, PB-S, and JS have contributed to the study conception and design, analyses and interpretation of the data, draft of the article, and final approval of the version to be published in accordance with all aspects of the work. VS, BR-V, TC, MC, RB, MT, GVS, LZ, TT-M, GS, and LF have contributed to the data acquisition and analysis, draft of the manuscript, and final approval of the version to be published in accordance with all aspects of the work. All authors contributed to the article and approved the submitted version.

## Funding

This study was supported by grants from the São Paulo Research Foundation (FAPESP)—grant numbers: 2013/08242-3 and 2015/07727-9; Brazilian Coordination for the Improvement of Higher Education Personnel (CAPES)—financial code 001; National Council for Scientific and Technological Development (CNPq)—grant numbers: 311368/2019-0 and 44004/2014-5; National Institute of Health Research (NIHR CL); National Funding for Scientific and Technologic Development of Chile (FONDECYT)—grant number: 3210687; Universidad de La Frontera (DIUFRO)—grant number: DIM20-0019; and Universidad de La Frontera—grant number: VRIP20P002.

## Conflict of Interest

The authors declare that the research was conducted in the absence of any commercial or financial relationships that could be construed as a potential conflict of interest.

## Publisher’s Note

All claims expressed in this article are solely those of the authors and do not necessarily represent those of their affiliated organizations, or those of the publisher, the editors and the reviewers. Any product that may be evaluated in this article, or claim that may be made by its manufacturer, is not guaranteed or endorsed by the publisher.
